# A 3D-printed surgical guide for ischemic scar targeting and ablation

**DOI:** 10.3389/fcvm.2022.1029816

**Published:** 2022-11-18

**Authors:** Mara Candelari, Ida Anna Cappello, Luigi Pannone, Cinzia Monaco, Giacomo Talevi, Edoardo Bori, Robbert Ramak, Mark La Meir, Ali Gharaviri, Gian Battista Chierchia, Bernardo Innocenti, Carlo de Asmundis

**Affiliations:** ^1^Heart Rhythm Management Centre, Postgraduate Program in Cardiac Electrophysiology and Pacing, European Reference Networks Guard-Heart, Universitair Ziekenhuis Brussel–Vrije Universiteit Brussel, Brussels, Belgium; ^2^BEAMS Department (Bio Electro and Mechanical Systems), Université Libre de Bruxelles, Brussels, Belgium; ^3^Cardiac Surgery Department, Universitair Ziekenhuis Brussel–Vrije Universiteit Brussel, Brussels, Belgium

**Keywords:** patient-specific modeling, heart model, 3D heart surgical guide, 3D printing, ischemic scar ablation treatment

## Abstract

**Background:**

3D printing technology development in medical fields allows to create 3D models to assist preoperative planning and support surgical procedures. Cardiac ischemic scar is clinically associated with malignant arrhythmias. Catheter ablation is aimed at eliminating the arrhythmogenic tissue until the sinus rhythm is restored. The scope of this work is to describe the workflow for a 3D surgical guide able to define the ischemic scar and target catheter ablation.

**Materials and methods:**

For the patient-specific 3D surgical guide and 3D heart phantom model realization, both CT scan and cardiac MRI images were processed; this was necessary to extract anatomical structures and pathological information, respectively. Medical images were uploaded and processed in 3D Slicer. For the surgical guide modeling, images from CT scan and MRI were loaded in Meshmixer and merged. For the heart phantom realization, only the CT segmentation was loaded in Meshmixer. The surgical guide was printed in MED625FLX with Polyjet technology. The heart phantom was printed in polylactide with FDM technology.

**Results:**

3D-printed surgical model was in agreement with prespecified imputed measurements. The phantom fitting test showed high accuracy of the 3D surgical tool compared with the patient-specific reproduced heart. Anatomical references in the surgical guide ensured good stability. Ablation catheter fitting test showed high suitability of the guide for different ablation tools.

**Conclusion:**

A 3D-printed guide for ventricular tachycardia ablation is feasible and accurate in terms of measurements, stability, and geometrical structure. Concerning clinical use, further clinical investigations are eagerly awaited.

## Introduction

In the past years, the rapid development of the 3D printing technology in many different medical fields like orthopedic, dentistry, and cardiovascular systems allowed to create 3D models for several purposes. The model building starts with medical imaging data acquisition, like CT and MRI.

Indeed, the 3D-reconstructed models aim at replicating anatomical and pathological structures to assist preoperative planning and simulate surgical or interventional procedures; furthermore, they can be useful to achieve precision medicine for improvement of treatment outcomes and provide medical education (1, 2).

**FIGURE 1:**
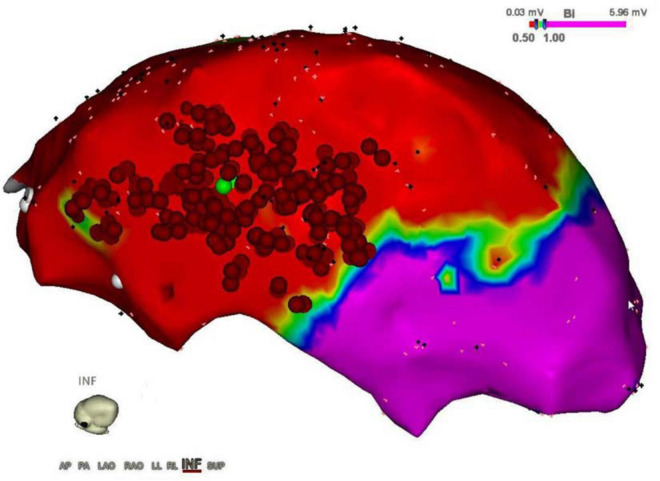
Epicardial electroanatomic mapping. The picture shows the epicardial electroanatomic mapping of both ventricles from the inferior view: voltages below 0.5 mV (red color) were considered scar and voltages above 1.0 mV were considered normal myocardium (purple color). In between border zone, tissue is colored yellow, green, and blue. Ablation points are represented by the red dots, performed to eliminate ventricular tachycardia.

The treatment of ischemic scar in patients post-myocardial infarction (MI) is clinically important because ischemic scar leads to malignant reentrant arrhythmias like ventricular tachycardia (VT), which represents a common cause of morbidity and mortality. Nowadays, one of the treatments consists of VT ablation that is aimed at eliminating the tissue (substrate) responsible for the arrhythmia. During VT catheter ablation, electrical activity mapping, which identifies the cardiac scar (target), is followed by the ablation of the arrhythmogenic tissue until the sinus rhythm is restored. Nevertheless, the lack of visual references for the pathological area to target could lead to incomplete ablation of arrhythmogenic substrate with subsequent recurrence of arrhythmias (3, 4). This might be an issue during open-chest, hybrid or thoracoscopic epicardial ablation. An example of an epicardial electroanatomic mapping and successful ablation of VT is shown in Figure 1.

A surgical guide may overcome these limitations, providing a reliable tool to properly define the area of interest for ablation. To build a surgical guide, it is necessary to consider surgeon’s and engineer’s requirements in terms of structure, shape, efficiency, and manufacturing time.

This article aims to define in detail the steps for a surgical guide realization to offer a practical and methodological guide, operatively pointing out the workflow that, from a CT scan and cardiac MRI imaging (CMR) acquisition, leads to the creation of patient-specific heart model using 3D printing technology. In particular, the purpose of this work is to build a cardiac surgical guide able to clearly indicate, directly on the heart surface, the pathological area to be treated. Such a guide can enable an improved solution for ischemic scar treatment, reducing the medical procedure timing, and enhancing the treatment outcomes.

## Materials and methods

### Patient selection

In this study, a 61-year-old man was included. In addition to presenting different cardiovascular risk factors, the patient had a previous lateral MI. At CMR, there was evidence of delayed enhancement with transmural late gadolinium enhancement (LGE), infarction of papillary muscles, and a severely dilated left ventricle with severely reduced ejection fraction and akinesia at the lateral wall from base to apex. The patient had recurrent monomorphic VT resistant to medical therapy, leading to multiple appropriate ICD shocks. Therefore, VT ablation was deemed indicated by the heart team, making the patient an appropriate candidate for this study. The study complied with the Declaration of Helsinki as revised in 2013; the ethical committee approved the study. The patient signed an informed consent form.

### Data acquisition

**FIGURE 2:**
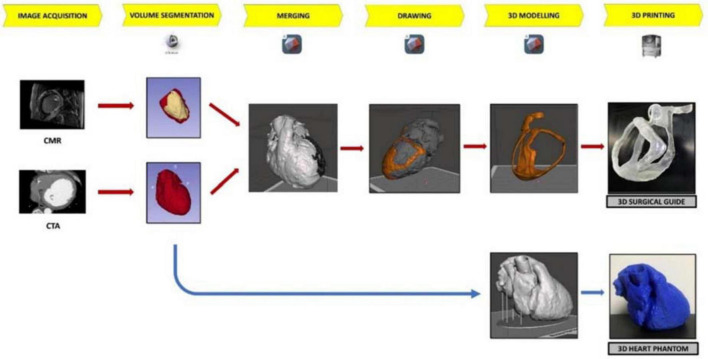
The workflow of 3D surgical guide and the 3D heart phantom. The workflow describes the main steps of surgical guide and heart phantom development process from the acquisition data to 3D printing.

For the aim of this study, both a surgical guide for VT ablation and a patient-specific heart phantom model for fitting test were built (Figure 2). Patient-specific CT and CMR were processed for the segmentation to extract anatomical structures and pathological information, respectively; CMR was performed with Philips Ingenia 1.5 T MR system (Koninklijke Philips N.V. Netherlands) at 1.5 T of magnetic field, at 63 MHz of imaging frequency, and 2.0 ms of repetition time. Cine CMR was recorded in transversal orientation with 360 slices of 288 × 288 pixels, at 10.0 mm of thickness. Instead, contrast CT scan (GE Healthcare, Chicago, IL, USA) was performed at 256 slices of 6.0-mm thickness, with 512 × 512 pixels. After acquisition, all data in Digital Imaging and Communications in Medicine format were stored in the Picture Archiving and Communication System.

### Segmentation and reconstruction of the 3D surgical guide model

To generate a patient-specific 3D model for surgical guide of VT ablation, two types of medical images were uploaded and separately processed in 3D Slicer (Surgical Planning Laboratory of Brigham and Women’s Hospital, Boston, MA, USA):

• CMR, for the target area identification and
• CT images as a template for the anatomy of the surgical guide model.

The CT images allowed the anatomical reconstruction of the guide model because of the reasonable soft-tissue contrast and the excellent spatial resolution necessary to draw and define the surgical guide, with the specific patient’s heart surface (5). The CT files also provided the necessary volumetric data required to create the patient-specific heart phantom for the fitting test.

In contrast, CMR was used for the identification of the arrhythmic substrate by means of the steady-state free precession cine CMR and LGE imaging. Indeed, the steady-state free precession cine technique provides dynamic heart visualization during the full cardiac cycle; the LGE technique is based on the slow washout of the gadolinium by the fibrotic tissue in contrast to the normal myocardium (6).

**FIGURE 3:**
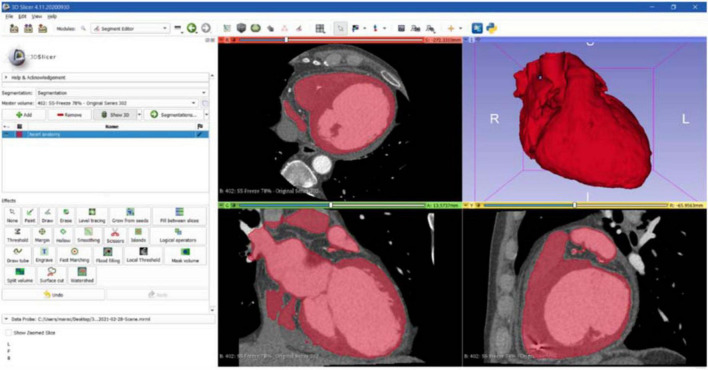
CT segmentation. Axial, sagittal, coronal, and 3D views of CT segmentation on the 3D Slicer software.

**FIGURE 4:**
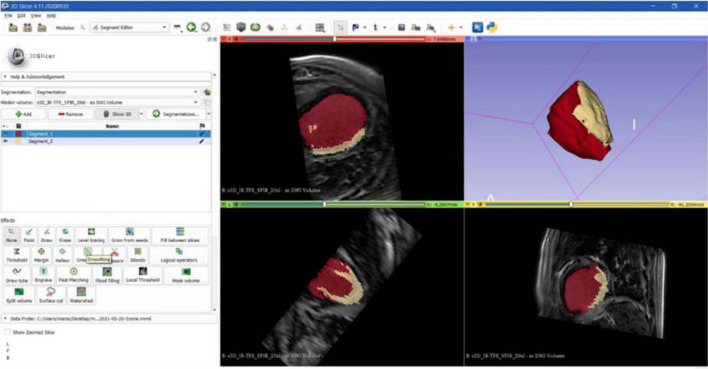
CMR segmentation. Axial, sagittal, coronal, and 3D views of CMR segmentation on the 3D Slicer software: the yellow zone represents the target area (scar), the red one represents the healthy tissue.

The first step to create a 3D reconstruction from the medical images was the segmentation, a semiautomatic process, which divides the image into regions with similar properties such as gray level, brightness, and contrast. For this study, the threshold-based segmentation was implemented to separate the anatomical region of interest (ROI) from the background by setting a threshold range, which identifies the cardiac tissue. Regarding the CT scan, even though the 3D Slicer software provided a specific threshold for cardiac tissue, the semiautomatic threshold-based segmentation could not be accurate enough because of anatomical structure heterogeneity in size, shape, and location and because of streak artifacts, metal implants, or boundaries ambiguity due to a low contrast between target organs and the neighboring tissue (7). Thus, the threshold for the CT model was manually arranged from 1 to 375, while for the CMR segmentation the range threshold was set from 99 to 509 in order to include the whole ROI (Figures 3, 4, respectively).

Furthermore, a supervised slice-by-slice segmentation through operator interaction was used for error correction in the event of an inadequate result by manually deleting unwanted regions. Thereafter, the postprocessing step was performed to smooth the surface to close undesired holes and remove distortions due to noisy acquisition.

To ensure the 3D-reliable reconstruction from CT scan, the heart anatomy was segmented into two parts, namely, cardiac tissue and internal chambers volume separately, which were later combined in an assembly.

Successively, the CMR, performed by using the different LGE intensity contrast, was manually segmented by distinguishing the pathological area (presence of LGE) from the rest of the heart. The result was a single 3D model with a target ROI (scar border identification). This represents a crucial step because in this phase the surgical opening in the guide is created to target the pathological area. The surgical hole is planned according to clinical considerations and LGE by an experienced cardiologist, and it is the definitive target area of the 3D surgical guide.

At the end of this process, two 3D heart models were generated and converted into STL format files. In particular, the 3D heart reconstruction generated from CT images was used as a template for the surgical guide surface model, and the other file represented the target area to be treated.

**FIGURE 5:**
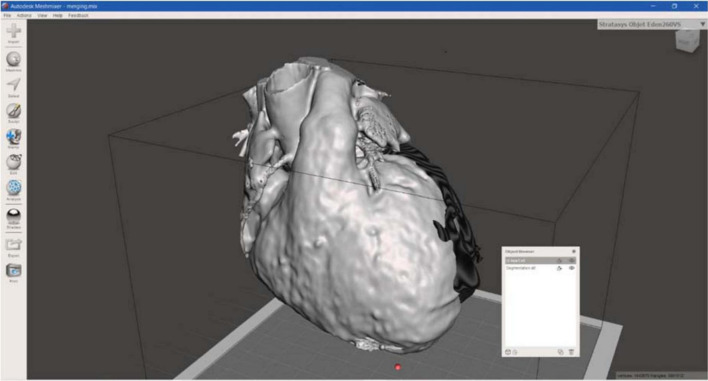
3D models merging. CT and CMRI models merging on the Autodesk Meshmixer software.

Regarding the surgical guide modeling, both 3D models from CT and CMRI were loaded in Meshmixer, Autodesk Inc., San Rafael, CA, USA, and by using the left ventricle and the apex, as anatomical landmarks, the merging of the models was achieved (Figure 5).

Then, the CT heart surface was used as a template to achieve a surgical guide characterized by the patient-specific heart surface. First, the scar border was drawn, and then the anatomical landmarks, such as the aorta ring, the pulmonary artery annulus, and heart apex were drafted and manually connected to the pathological target area. Other reference points represented by the main coronary arteries were added, creating the whole surgical guide surface.

Successively, the surgical guide sheet was extruded to have a 3D shape of 3.0 mm of thickness. In addition, for a better identification of the target area and a higher protection to healthy tissue from surgical procedure, an extra thickness of 3.0 mm around the target area was added, reaching 6.0 mm of thickness. These thicknesses were defined according to use considerations: 3.0 mm allows to maintain flexibility and lightweight, avoiding damages during handling, and the extra border was useful to identify the target to be treated.

**FIGURE 6:**
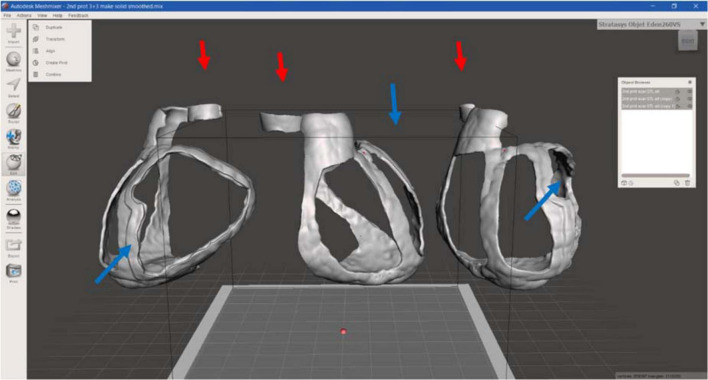
3D surgical guide model. Anterior, lateral, and posterior view of the virtual surgical guide model: the blue arrows indicate the thicker border for the target (scar) identification, the red ones the reference point for aorta.

Moreover, to improve the surface design the “brush” function was performed and the whole guide model was subjected to smoothing phase. Finally, the 3D surgical guide model was converted into STL format to create a suitable file to print. Figure 6 represents the 3D model of surgical mask from different views.

**FIGURE 7:**
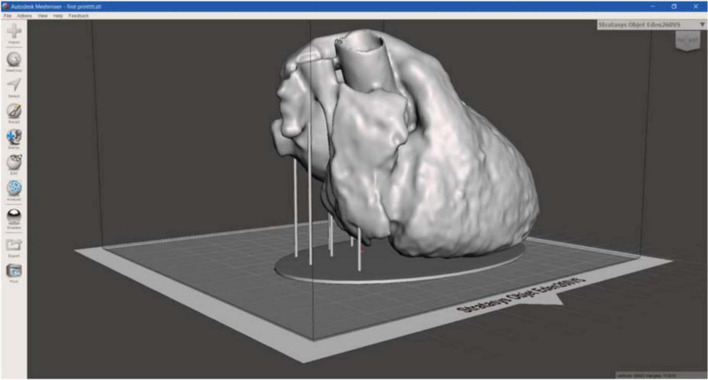
3D heart phantom model. 3D model of heart phantom with additional components, such as the base and the vertical cylindrical structures, which connects the base to the heart, to stabilize the model, useful to avoid the collapse during printing.

For the heart phantom realization, the CT segmentation was loaded in the Meshmixer software to create structural additional components, necessary to stabilize the model to be printed, avoiding the collapse (Figure 7).

### 3D surgical guide printing

For this step, two types of 3D printers were used: (1) the Stratasys Objet260 Connex1 (Stratasys, USA), based on Polyjet technology, with MED625FLX to create the 3D surgical guide; and (2) the PRUSA imk3 3D printer (Josef Prusa, Prague, Czech Republic), based on FDM technology, with polylactide (PLA), for the heart phantom. Once the STL file of the 3D surgical guide was ready, it was imported in the Object Studio software (the software provided by Stratasys for printing settings), where it was automatically displaced on the virtual tray, and the printing was started.

Stratasys Objet260 Connex1 is a Polyjet 3D printing, an additive manufacturing process in which layers of polymeric materials are jetted at specific position onto a build tray and the UV lamps, mounted on the print block, partially drying the material on each pass. The print block travels from right to left, and then, the Z-stage moves upward slightly, creating the 3D object. During printing, the 3D model was inserted into the matrix, the GelMatrix, also called Support material, an alkaline solution-soluble material utilized when printing small features. The GelMatrix material is softer and easier to remove; it allows to realize a small diameter structure previously unattainable by 3D printing and gives enough support during the printing process (8).

The whole 3D prototype was printed by Stratasys Objet260 Connex1 in MED625FLX, a biocompatible polymeric material provided by the Stratasys, characterized by transparence and lightweight, and the SUP705 was used as GelMatrix. After printing, the 3D model was washed in a specific support material removal system, provided by Stratasys: the WaterJet, a compact and affordable system able to efficiently clean any type of 3D model or prototype. During washing, it is important to verify the part is fully cleaned to enable its biocompatibility properties because support material is not biocompatible. It is best to hold the waterjet nozzle at safe distance from the model—approximately 20.0 cm is considered safe. It is also recommended to move the nozzle with quick movements (not fixed on a single location for more than 2.0 s) to avoid damaging due to high water impact (9).

When all settings were selected, the printing time was calculated by the software, accounting for the object position on the tray.

### 3D surgical guide testing

After printing, 3D surgical guide was measured to evaluate its consistency with prespecified imputed measurements. To assess the anatomical accuracy and stability of the surgical guide, a patient-specific heart phantom fitting test was performed. Finally, to test the suitability of the guide for ablation, an ablation catheter fitting test was performed. This included different ablation tools as follows: (1) cryoablation probe (10-cm-aluminum CryoICE Cryoablation Probe, AtriCure Inc., OH, USA); (2) radiofrequency 3.5-mm tip catheter (3.5-mm tip ablation catheter, FlexAbility, St. Jude Medical, Abbott Park, IL, USA); and (3) bipolar radiofrequency linear catheter (bipolar unidirectional RF linear device, Coolrail, AtriC Inc., West Chester, OH, USA).

## Results

The printing time was approximately 10 h and 50 min, with a consumption of MED625FLX of approximately 247.0 g and 556.0 g of support material. All these data are reported in Table 1. The final surgical guide had the following measurements (137.7 mm × 141.9 mm × 119.6 mm), in agreement with prespecified imputed measurements. Moreover, to determine the accuracy of 3D printing, thickness was measured, which was 3.0 mm for the whole model and 6.0 mm for the target area border, as expected.

**TABLE 1:** Main features of 3D surgical guide printing.

Material consumption	247.0 g
Support material consumption	556.0 g
Printing estimation time	10 h 50′

The 3D surgical guide was characterized by a thicker border around target area, directly indicating the pathological area. For a correct visualization and positioning on the heart surface, the surgical guide presented different reference points, such as the aorta ring, the pulmonary artery annulus, and the apex, connecting each other following part of the main coronary arteries, resulting in an additional reference point for the surgeon. Both internal and external surfaces were regular and smooth also because of the “Waterjet,” which allowed a perfect cleaning, without any visible trace of support material.

**FIGURE 8:**
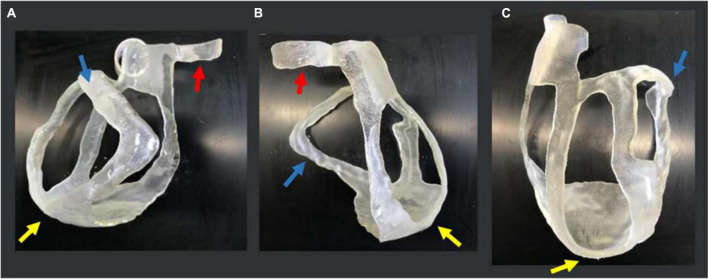
3D-printed surgical guide prototype. Three views of the physical 3D surgical guide: **(A)** antero-posterior view; **(B)** latero-lateral view; and **(C)** postero-anterior view. The blue arrows indicate the thicker border for the target (scar) identification, the yellow ones indicate the apex, and the red ones the aorta annulus necessary for the application and positioning on the heart.

The 3D-printed surgical guide is represented in three different views in Figure 8.

**FIGURE 9:**
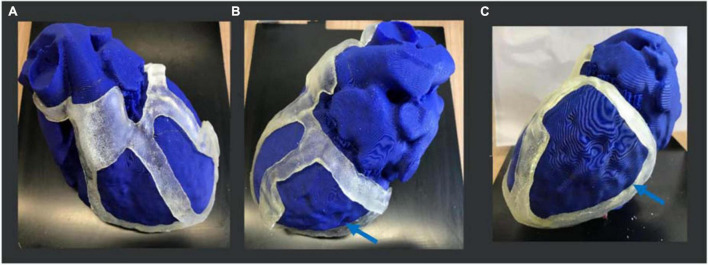
3D heart phantom fitting test. Three views of the physical 3D surgical guide on the phantom: **(A)** antero-posterior view; **(B)** latero-lateral view; **(C)** postero-anterior view. The 3D surgical guide fitting simulation is performed on the heart phantom, previously printed in PLA by PRUSA 3D printer. The blue arrows show the target (scar) area to be treated.

For the heart phantom printing, the STL file was sent to PRUSA and the model was realized in 15 h. The heart phantom fitting test confirmed the anatomical accuracy and stability of the surgical guide. The surgical guide was placed on the phantom, and it was fitted with the patient-specific heart model. The reference points like the aorta, pulmonary artery rings, and apex ensured good stability (Figure 9).

**FIGURE 10:**
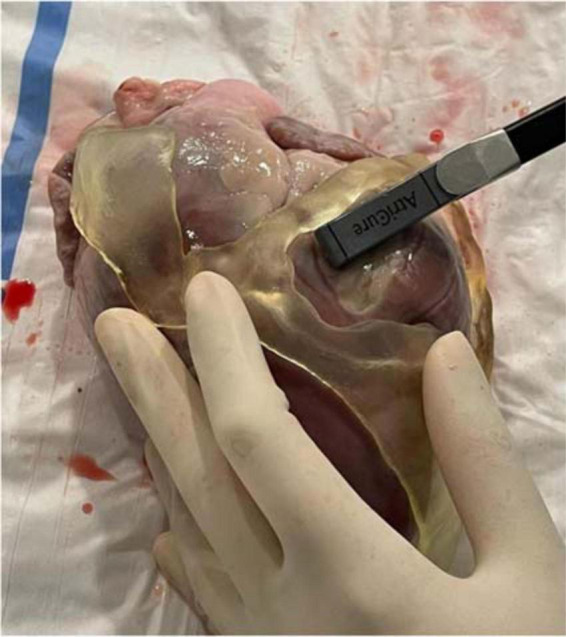
Ablation catheter fitting test. Ablation catheter fitting test showed high suitability of the guide for different ablation tools, such as the bipolar radiofrequency linear catheter in the figure (bipolar unidirectional RF linear device, Coolrail, AtriC Inc., West Chester, OH, USA). All catheters could be easily positioned and manipulated within the surgical guide.

Ablation catheter fitting test showed high suitability of the guide for different ablation tools including (1) cryoablation probe (10-cm-aluminum CryoICE Cryoablation Probe, AtriCure Inc., OH, USA); (2) radiofrequency 3.5-mm tip catheter (3.5-mm tip ablation catheter, FlexAbility, St. Jude Medical); and (3) bipolar radiofrequency linear catheter (bipolar unidirectional RF linear device, Coolrail, AtriC Inc., West Chester, OH, USA). All catheters could be easily positioned and manipulated within the surgical guide (Figure 10).

## Discussion

The main results of this study are (1) a 3D-printed surgical guide for VT ablation is feasible; (2) based on the fitting test results on the heart phantom, a 3D-printed surgical guide is accurate; and (3) different ablation catheters can be easily positioned and manipulated within the surgical guide.

In the past decades, the 3D printing technology development in medical fields allowed to create 3D models, aiming to replicate anatomical and pathological structures, assist preoperative planning, and support surgical procedures (1, 2). It overcomes some of the limitations of conventional 2D/3D imaging methods by providing a tangible, physical 3D model of complex anatomical structures. 3D models have shown promising results in widespread applications from education to procedural planning and device testing (10). Most of the 3D models described in the literature are designed as an aid in the preprocedural planning. The model hereby presented is aimed at directly helping the surgeon to identify the ischemic scar area during ablation.

The concept of a patient-specific surgical guide for personalized epicardial ischemic scar treatment in patients post MI derived from the need to have a precise visualization of pathological area to assess the necrotic extent of ablation lesions; indeed, an incomplete ablation of arrhythmogenic substrate can cause subsequent recovery of tissue with restoration of conduction and recurrent arrhythmias.

The purpose of this work was to apply the additive manufacturing technique to create a 3D model, able to easily identify the target area, previously planned by the cardiologist; the model is designed to be used on the epicardium of the heart, during cardiac surgery, reducing the medical procedure timing and improving the treatment results.

The high-resolution imaging from CMR and CT, the Computer-Aided Design (CAD) software and the current 3D printing technology allow the development and realization of the 3D surgical guide for a precise identification of ischemic scar; the target zone was designed during the segmentation phase, and it can be tailored according to patient characteristics, clinical considerations, and treatment planning.

Indeed, in recent years, cardiac imaging (CT scan, CMR, intracardiac echocardiography) has emerged as a valuable tool in planning and guiding VT ablation. Cardiac images can be merged with the intraprocedural mapping acquired by mapping or ablation catheter. The potential benefit of a 3D-printed guide is the possibility to use it in epicardial-only procedure (open heart surgery or thoracoscopic) to delineate the area of interest of the VT circuit (scar and border zone). The information from CT scan and CMR is already integrated into the guide, potentially not requiring mapping (e.g., a surgical guide may represent an added value in case of non-inducible or non-hemodynamically tolerated VT). This does not prevent or exclude activation mapping and entrainment of the VT, which instead might be prompted by anatomical delineation.

The 3D-printed surgical guide prototype is represented by a patient-specific heart structure, characterized by links, ensuring stability, with moderate consumption of material and manufacturing time.

The anatomical landmarks like the aorta, pulmonary artery, and the apex, together with the heart surface shape, ensuring stability, allow a rapid and easy application and fitting on the heart. A proper flexibility is given by the polymeric material with a thickness of approximately 3.0 mm in the body and 6.0 mm in the target area border.

Therefore, this study indicates that a 3D surgical guide is a reliable technology that might contribute to a rapid and more precise identification of the pathological area during epicardial VT ablation.

Further studies are needed to evaluate reproducibility and timing of the proposed workflow. Indeed, 3D printing is a relatively long process. It can be completed in approximately 10 h. 3D printing of a surgical guide will be integrated into a procedural workflow for VT ablation starting with (1) obtaining CT scan and CMR (possible also 1–2 weeks before ablation); (2) analyzing and segmenting both CT and CMR; (3) creating the model of the surgical guide; (4) printing and first cleaning of the guide; and (5) second cleaning and sterilization phase, not earlier than 24 h before the ablation. The estimated time for modeling and printing of 3D surgical guide is approximately 48–72 h (that is compatible with real-world clinical practice).

In addition to reproducibility, another limitation is represented by segmentation as a supervised slice-by-slice segmentation through operator interaction is required. This is necessary because of boundaries ambiguity due to a low contrast between target organs and the neighboring tissue (5). Automatic and standardized image segmentation process is eagerly awaited to decrease costs of personnel and allow more reproducible results. The cost of a surgical guide is mostly dependent on the biomaterial and the support material used. Furthermore, the guide being patient-specific, the amount of material used and costs depend on patient’s heart. In the presented case, 117 g of biomaterial and 180 g of support material have been used, for a total amount of ≈80 €.

Finally, further tests to evaluate the response of materials to ablation energy sources are awaited. In particular, both materials have been recently tested by our group with radiofrequency catheter ablation (11). For surgical guides design, the 2.5-mm MED625FLX could be used, with bipolar radiofrequency catheter, ensuring good geometrical, mechanical, and thermal properties. None of the two biomaterials tested is suitable for unipolar radiofrequency ablation.

## Conclusion

A 3D-printed surgical guide for epicardial VT ablation is feasible and geometrically accurate. It can contribute to a rapid and more precise identification of the pathological area. Further investigations are needed, applying these models to material validation tests and *in vitro* testing to assess the material properties and the biocompatibility in response to the surgical tools used for the ablation treatment.

## Data availability statement

The original contributions presented in this study are included in the article/supplementary material, further inquiries can be directed to the corresponding author/s.

## Ethics statement

The studies involving human participants were reviewed and approved by Commissie Medische Ethiek, UZ Brussel. The patients/participants provided their written informed consent to participate in this study.

## Author contributions

MC, BI, and CA: conception and design of the work. MC, IC, LP, CM, GT, and RR: substantial contributions to the acquisition of data for the work. MC, LP, EB, and GT: substantial contributions to the analysis of data for the work. RR, ML, AG, GC, BI, and CA: substantial contributions to the interpretation of data for the work. MC and IC: drafting the work. GT, RR, ML, AG, GC, BI, and CA: revising the draft of the work critically for important intellectual content. All authors final approval of the version to be published and agreement to be accountable for all aspects of the work in ensuring that questions related to the accuracy or integrity of any part of the work were appropriately investigated and resolved.
